# Supplementary material for: Young Plasma Rejuvenates Blood DNA Methylation Profile, Extends Mean Lifespan, and Improves Physical Appearance in Old Rats
Source: J Gerontol A Biol Sci Med Sci. 2024 Mar 2;79(5):glae071. doi: 10.1093/gerona/glae071 (PMC11020299; doi:10.1093/gerona/glae071)
Supplement: glae071_suppl_Supplementary_Material [file glae071_suppl_supplementary_material.zip › Suppl Table 1-5/Supp Table Captions 1-5.docx]

**Supplemental Table 1-** Gene annotation of positively differentially methylated probes (qval<0.05).

**Supplemental Table 2-** Gene annotation of negatively differentially methylated probes (qval<0.05).

**Supplemental Table 3-** Gene annotation of positively differentially methylated CpGs present in promoters (qval<0.05).

**Supplemental Table 4-** Gene annotation of negatively differentially methylated CpGs present in promoters (qval<0.05).

**Supplemental Table 5-** Functional enrichment of differentially methylated genes in promoter regions of blood DNA from control and treated rats.
